# Q-Learning-Based Pending Zone Adjustment for Proximity Classification

**DOI:** 10.3390/s23094352

**Published:** 2023-04-28

**Authors:** Jung-Hyok Kwon, Sol-Bee Lee, Eui-Jik Kim

**Affiliations:** 1 Smart Computing Laboratory, Hallym University, 1 Hallymdaehak-gil, Chuncheon 24252, Gangwon-do, Republic of Korea; jhkwon@hallym.ac.kr; 2 Division of Software, Hallym University, 1 Hallymdaehak-gil, Chuncheon 24252, Gangwon-do, Republic of Korea; thfqla3535@hallym.ac.kr

**Keywords:** pending zone, proximity classification, proximity-based services, received signal strength indicator, Q-learning

## Abstract

This paper presents a Q-learning-based pending zone adjustment for received signal strength indicator (RSSI)-based proximity classification (QPZA). QPZA aims to improve the accuracy of RSSI-based proximity classification by adaptively adjusting the size of the pending zone, taking into account changes in the surrounding environment. The pending zone refers to an area in which the previous result of proximity classification is maintained and is expressed as a near boundary and a far boundary. QPZA uses Q-learning to expand the size of the pending zone when the noise level increases and reduce it otherwise. Specifically, it calculates the noise level using the estimation error of a device deployed at a specific location. Then, QPZA adjusts the near boundary and far boundary separately by inputting the noise level into the near and far boundary adjusters, consisting of the Q-learning agent and reward calculator. The Q-learning agent determines the next boundary using the Q-table, and the reward calculator calculates the reward using the noise level. QPZA updates the Q-table of the Q-learning agent using the reward. To evaluate the performance of QPZA, we conducted an experimental implementation and compared the accuracy of QPZA with that of the existing approach. The results showed that QPZA achieves 11.69% higher accuracy compared to the existing approach, on average.

## 1. Introduction

Recently, proximity-based service (PBS) has been widely used in various industries, including agriculture, commerce, construction, education, and healthcare, due to its operational simplicity and infrastructure-less feature [1,2,3]. In these industries, PBS has been utilized to support a variety of applications, such as inventory management, advertisement, safety management, attendance management, and patient tracking [4,5,6]. Especially during the COVID-19 pandemic, PBS was used for contact tracing to identify close contact with an infected person within six feet, making it one of the promising solutions to prevent the spread of the coronavirus [7,8]. Traditionally, location-based services (LBS) have been used to support applications where a central server estimates the exact position (i.e., coordinates) of devices using trilateration or fingerprinting schemes [9]. However, LBS has higher complexity with respect to the system architecture and therefore requires higher installation and maintenance costs compared to PBS. Unlike LBS, PBS has a simple system architecture consisting of a transmitter and receiver pair, and provides a simple operational procedure. Specifically, the receiver in PBS directly estimates the relative distance between the transmitter through direct communication channels such as Bluetooth and Wi-Fi Direct.

In general, the received signal strength indicator (RSSI) is used to estimate the distance between devices for proximity classification. Specifically, a receiver measures the RSSI of the signal transmitted by a sender and estimates the distance from the sender using a log-distance propagation model that characterizes the relationship between the RSSI and the distance [10,11]. The estimated distance is then compared with the distance between the receiver and the reference boundary (i.e., reference distance) predefined for proximity classification. If the estimated distance is shorter than the reference distance, the receiver decides that the sender is adjacent; otherwise, it decides that the sender is far away. However, in real-world environments, the RSSI changes frequently, even if the distance between the sender and receiver is fixed. This is because the RSSI is easily affected by the multi-path fading caused by the reflection and diffraction of radio signals and the interference by radio signals transmitted via the same frequency band [12,13]. The fluctuation in the RSSI makes accurate proximity classification difficult, and therefore there are numerous studies on improving the accuracy of RSSI-based proximity classification.

To address this problem, most studies have focused on minimizing the error of distance estimation in RSSI-based proximity classification. Mathematical models (e.g., modified log-distance models), statistical models (e.g., moving average models), filtering models (e.g., Kalman filter models), and machine learning models (e.g., linear regression models) were used in [14,15,16,17] to minimize estimation errors. However, improving the distance estimation model alone may not be sufficient to guarantee the accuracy of RSSI-based proximity classification, as even sophisticated distance estimation models have estimation errors. In particular, when the actual distance between the sender and receiver is close to the reference boundary, the result of RSSI-based proximity classification may frequently change, even if the actual distance is fixed. To overcome this problem, authors in [18,19] used a pending zone that surrounded the reference boundary. The use of a pending zone mitigates frequent changes in the results of proximity classification when the actual distance between the sender and receiver is close to the reference boundary because, within the pending zone, the previously estimated distance is maintained instead of a newly estimating the distance. However, the approaches proposed in [18,19] may suffer from low accuracy since they use a fixed size for the pending zone, regardless of the estimation error that varies depending on the surrounding environment. Therefore, to improve accuracy, it is necessary to adaptively adjust the size of the pending zone according to changes in the surrounding environment.

In this paper, we propose Q-learning-based pending zone adjustment for RSSI-based proximity classification (QPZA). QPZA runs on the BLE tag receiver to improve accuracy by adaptively adjusting the size of the pending zone, considering changes in the surrounding environment. Specifically, QPZA uses Q-learning to expand the size of the pending zone when the noise level increases and reduce it otherwise. Q-learning is a model-free reinforcement learning technique in which the Q-learning agent seeks the best action for the next state, considering the cumulative reward. In QPZA, the action entails adjusting the size of the pending zone, and the reward indicates the suitability of the action for the current noise level. The state refers to the distance between the receiver and the boundary of the pending zone. The size of the pending zone is the area between two decision boundaries, i.e., that between the near boundary and the far boundary. Therefore, QPZA employs two Q-learning agents to separately adjust the near boundary and far boundary. To determine the number of states for each Q-learning agent, we use a mean-shift clustering algorithm to cluster the noise level dataset and calculate the value of each state considering the centroid for each cluster. QPZA uses two different reward functions to individually set the reward for the near boundary and far boundary. To evaluate the performance of QPZA, we conducted an experimental implementation and compared it with the existing approach that uses a fixed size of the pending zone. The results showed that QPZA has 11.69% higher accuracy compared to the existing approach, on average.

The rest of this paper is organized as follows: Section 2 presents the system model, which includes the system architecture and distance estimation model. In Section 3, we describe the design of QPZA in detail. In Section 4, the results of the implementation and performance evaluation are presented. Finally, in Section 5, we conclude this paper.

## 2. System Model

QPZA is designed to improve the accuracy of RSSI-based proximity classification using Q-learning. In the following subsections, we first describe the architecture of the RSSI-based proximity classification system with QPZA. It is assumed that devices communicate with each other using Bluetooth low energy (BLE). Thus, the system consists of senders (i.e., BLE tags), a reference device (i.e., an anchor beacon), and a receiver (i.e., a BLE tag receiver). Then, we present the linear regression-based distance estimation model that is used to estimate the distance between the BLE tag receiver and the BLE tag.

### 2.1. System Architecture

**Figure 1:**
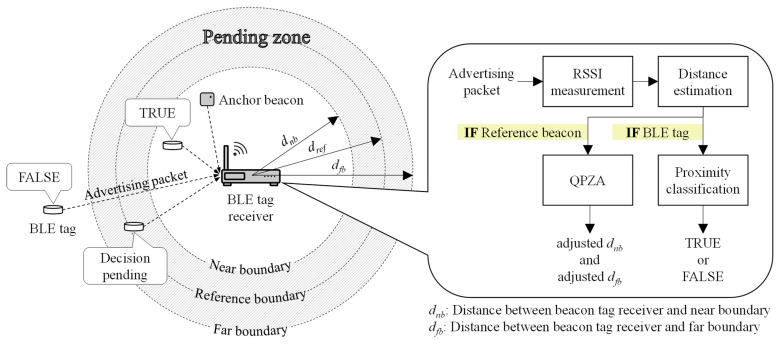
System architecture.

Figure 1 shows the architecture of the RSSI-based proximity classification system with QPZA. In the figure, the system is composed of BLE tags, an anchor beacon, and a BLE tag receiver. The BLE tags are responsible for broadcasting advertising packets to notify the BLE tag receiver of the beacon information, including a universal unique identifier (UUID), major ID, minor ID, and transmission power (TxPower). The UUID, major ID, and minor ID are used to identify the BLE tag, and the TxPower is used to estimate the distance between the identified BLE tag and the BLE tag receiver. The anchor beacon is a special type of BLE tag that is placed at a specific location. The anchor beacon broadcasts advertising packets in the same way the BLE tag does. However, the beacon information of the anchor beacon is used only to measure the noise level of the surrounding environment. Whenever the BLE tag receiver receives an advertising packet, it measures the RSSI and conducts distance estimation using the measured RSSI. To thoroughly examine the effect of adjusting the pending zone on the accuracy of RSSI-based proximity classification, we make the assumption that the distance estimation is based on the raw values of the measured RSSI. If the packet is received from the BLE tag, the BLE tag receiver conducts proximity classification to decide whether or not the BLE tag is nearby. In the other case, it calculates the noise level and conducts QPZA to adjust the size of the pending zone.

In the figure, the pending zone is illustrated as the area between the near boundary and the far boundary. The pending zone contains the reference boundary predefined by the system. Therefore, the distance between the BLE tag receiver and the near boundary ($d_{nb}$) is less than or equal to the distance between the BLE tag receiver and the reference boundary ($d_{ref}$), while the distance between the BLE tag receiver and the far boundary ($d_{nf}$) is greater than or equal to $d_{ref}$. For proximity classification, the BLE tag receiver determines that the BLE tag is adjacent (i.e., true) when the estimated distance of the BLE tag is less than $d_{nb}$. Conversely, it determines that the BLE tag is not adjacent (i.e., false) when the estimated distance of the BLE tag is greater than $d_{nf}$. If the estimated distance of the BLE tag is within the pending zone, the BLE tag receiver maintains the same results of proximity classification as the previous result (i.e., decision pending). In this context, $d_{nb}$ and $d_{nf}$ are the key criteria for proximity classification. In our work, QPZA adaptively adjusts $d_{nb}$ and $d_{nf}$, considering the noise level, thereby improving the accuracy of the proximity classification. In Section 3, we describe the design of QPZA in detail.

### 2.2. Distance Estimation Model

Upon receiving the advertising packet, the BLE tag receiver measures RSSI and estimates the distance using a distance estimation model. To this end, we consider three types of existing distance estimation models: the iBeacon, linear regression, and deep learning models [20,21,22]. Then, we select one of the models with the highest accuracy and apply it to the BLE tag receiver. In this subsection, we describe the design of each model in detail.

#### 2.2.1. iBeacon Model

The iBeacon model is provided by the iBeacon specification of Equation (1) [20].

(1) $$ed_{iBeacon}=0.89976{\left(\frac{RSSI}{\mathrm{TxPower}}\right)}^{7.7095}+0.111$$

where $ed_{iBeacon}$ is the estimated distance of the iBeacon model and RSSI is the value of the measured RSSI. The unit of RSSI is a dBm. In the equation, $ed_{iBeacon}$ is proportional to the RSSI and inversely proportional to the TxPower. The iBeacon model is built based on RSSI datasets that are pre-collected in specific environments. Therefore, it remains fixed regardless of changes in the environment.

#### 2.2.2. Linear Regression Model

To build the linear regression model, it is assumed that the relationship between RSSI and log-distance is given by Equation (2) [14].

(2) $$\log(d)=A\left(RSSI\right)+B$$

where d is the distance between the BLE tag receiver and the BLE tag, and A and B are the coefficient (i.e., regression slope) and log(d) intercept, respectively. The unit of d is meters. To determine parameters A and B in Equation (2), we use the log-distance dataset for RSSI-based distance estimation (LD), which is represented by Equation (3):

(3) $$LD=\left[\left(RSSI_{0},\log(d_{0})\right),\left(RSSI_{1},\log(d_{1})\right),\cdots ,\left(RSSI_{n},\log(d_{n})\right)\right]$$

where $RSSI_{n}$ is the value of the measured RSSI for the (n+1)-th advertising packet, and $d_{n}$ is the actual distance for the (n+1)-th advertising packet. Additionally, the ordinary least square is used to calculate parameters A and B [21]. Specifically, parameters A and B can be obtained by Equations (4) and (5), respectively:

(4) $$A=\frac{\sum_{i=0}^{n}\left(RSSI_{i}-\mathrm{mean}(RSSI)\right)\left(\log(d_{i})-\mathrm{mean}(\log(d))\right)}{\sum_{i=0}^{n}{\left(RSSI_{i}-\mathrm{mean}(RSSI)\right)}^{2}}$$

(5) $$B=\mathrm{mean}(\log(d))-{\left(\mathrm{mean}(RSSI)\times A\right)}^{2}$$

where mean(RSSI) is the average of every RSSI in LD, and mean(log(d)) is the average of every log(d) in LD. When obtaining parameters A and B, the distance estimation model expressed as Equation (6) is mounted into the BLE tag receiver. That is, whenever it receives an advertisement packet, the BLE tag receiver estimates the distance using the mounted distance estimation model.

(6) $$ed_{LR}=10^{\left(A(RSSI_{mean})+B\right)}$$

where $ed_{LR}$ is the estimated distance of linear regression model when the value of the measured RSSI is $RSSI_{mean}$. The linear regression model varies depending on the RSSI value, and thus it changes when the environment for RSSI collection changes.

#### 2.2.3. Deep Learning Model

**Figure 2:**
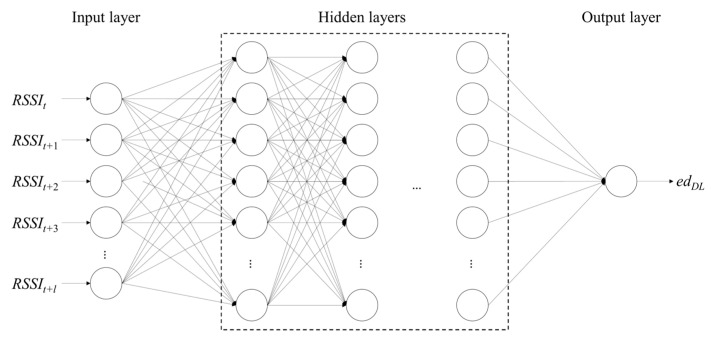
Shape of many-to-one DNN model.

To build a deep learning model, we first design a many-to-one deep neural network (DNN) model that uses multiple RSSI inputs to derive one estimated distance (i.e., $ed_{DL}$). Figure 2 shows the shape of the many-to-one DNN model, which consists of an input layer, hidden layers, and an output layer [22].

The input layer consists of multiple nodes (i.e., circles in the figure) to input the multiple RSSI inputs generated sequentially. In the figure, the number of RSSI inputs is equal to l. The number of hidden layers and the number of nodes in each hidden layer are empirically determined to obtain high accuracy. Additionally, we assume that the rectified linear unit (ReLU) is used as the activation function to solve the vanishing gradient problem, in which the gradient gradually converges to zero during the back-propagation process. Similar to the linear regression model, the deep learning model varies depending on the RSSI collection environment. However, unlike the other models, it requires multiple RSSI inputs to estimate distance.

## 3. Design of QPZA

QPZA is designed to adjust the size of the pending zone to improve the accuracy of proximity classification that varies according to the noise level of the surrounding environment. In QPZA, the BLE tag receiver first checks the noise level using the anchor beacon, and then it adjusts the size of the pending zone depending on the noise level. QPZA expands the size of the pending zone when the noise level increases. In this case, the difference between $d_{nb}$ and $d_{fb}$ increases. In the opposite case, the difference between $d_{nb}$ and $d_{fb}$ decreases.

**Figure 3:**
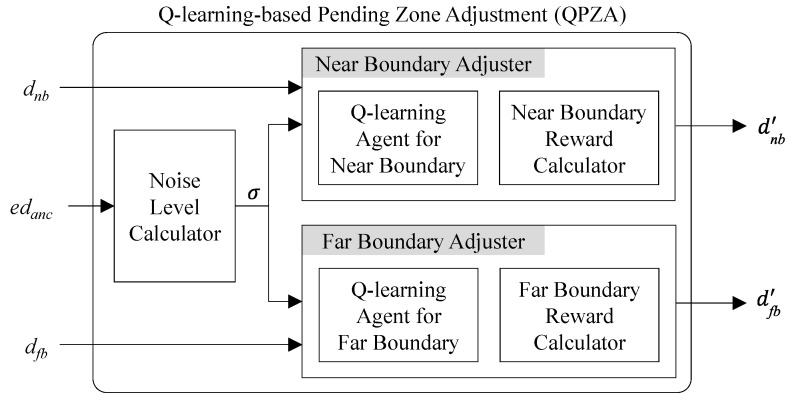
Operational block diagram for QPZA.

Figure 3 shows an operational block diagram for QPZA. In the figure, QPZA consists of a noise level calculator, a near boundary adjuster, and a far boundary adjuster. The noise level calculator is used to calculate the noise level (σ), and the near and far boundary adjusters are used to adjust $d_{nb}$ and $d_{fb}$, respectively. Each adjuster constitutes the Q-learning agent and the reward calculator. The former selects the action (i.e., decrease, keep, and increase) to determine the next $d_{nb}$ or $d_{fb}$ (i.e., ${d^{\prime }}_{nb}$ or ${d^{\prime }}_{fb}$). The latter checks whether or not the selected action is suitable for the noise level, and then calculates the reward considering the result of the check. The reward is used to update the Q-learning agent.

The BLE tag receiver conducts QPZA whenever it receives an advertising packet from the anchor beacon. Specifically, after obtaining the estimated distance of the anchor beacon (i.e., $ed_{anc}$), the noise level calculator runs to determine σ, which is calculated as the difference between $ed_{anc}$ and the actual distance between the BLE tag receiver and the anchor beacon (i.e., $d_{anc}$). Thus, in QPZA, the noise level is expressed in meters. Then, the near and far boundary adjusters adjust $d_{nb}$ and $d_{fb}$ considering the cumulative reward. To this end, the Q-learning agent for each adjuster maintains a Q-table. Table 1 and Table 2 show the Q-table for the near boundary (Q) and the Q-table for the far boundary ($\hat{Q}$), respectively.

In the Q-table, each row represents a candidate distance for each state, which can be selected as the distance between the BLE tag receiver and boundary. The state is the index of the candidate distance, and the number of states is equal to m for both boundaries. Accordingly, $d_{nb}$, ${d^{\prime }}_{nb}$, $d_{fb}$, and ${d^{\prime }}_{fb}$ can be given by Equations (7) and (8), respectively.

(7) $$d_{nb},{d^{\prime }}_{nb}\in \left\{nb_{1},nb_{2},\cdots ,nb_{m}\right\}$$

(8) $$d_{fb},{d^{\prime }}_{fb}\in \left\{fb_{1},fb_{2},\cdots ,fb_{m}\right\}$$

where $nb_{m}$ is the m-th candidate distance in the Q-table for the near boundary and $fb_{m}$ is the m-th candidate distance in the Q-table for the far boundary. The columns of the Q-tables represent the actions, including decrease, keep, and increase, and the index for each action is 1, 2, and 3, respectively. If the Q-learning agent decides the action to be decrease or increase, $d_{nb}$ and $d_{fb}$ change; otherwise, they are kept as their previous distance. The elements of the Q-table denote state-action values or Q-values, expressed as $Q(s_{nb},a_{nb})$ and $\hat{Q}(s_{fb},a_{fb})$ for near and far boundaries, respectively. For each boundary, $s_{nb}$ and $s_{fb}$ indicate the indices of the candidate distance (i.e., states), and $a_{nb}$ and $a_{fb}$ indicate the indices of the actions.

The number of states (i.e., m) is determined based on the number of clusters in the noise level dataset (NL), which consists of noise level data samples collected in an environment in which the number of neighboring BLE tags varies. The noise level dataset is given by Equation (9).

(9) $$NL=\left\{\sigma_{1},\sigma_{2},\cdots ,\sigma_{l}\right\}$$

where l is the number of noise level data samples in NL. To determine the number of clusters in NL, a mean-shift clustering algorithm is used. The algorithm creates a density function for a given dataset using kernel density estimation (KDE) and searches for local maxima in the density function [23]. The number of clusters is equal to the number of local maxima in the density function. The density function for NL can be obtained using KDE, as shown in Equation (10).

(10) $$f(x)=\frac{1}{lh}\sum_{i=1}^{l}K\left(\frac{x-\sigma_{i}}{h}\right)$$

where f(x) is the density function for NL, h is the bandwidth (also referred to as scale) for KDE, and K(x) is the Gaussian kernel function given by $K(x)=e^{-x/2}$. The value of h is determined to maximize the inter-cluster variance and minimize the inter-cluster variance. The number of local maxima in f(x) is obtained by counting the number of arguments (i.e., xs) that simultaneously satisfy $f^{\prime }(x)=0$ and $f^{\prime \prime }(x)<0$, where $f^{\prime }(x)$ and $f^{\prime \prime }(x)$ are the first and second derivatives of f(x), respectively. Note that x>0 and x∈ℝ. The value of the argument for each local maximum denotes the centroid of each cluster. Finally, m is determined as the sum of the number of local maxima and the number of reference boundaries (i.e., 1).

The value of the candidate distance for each state is determined using the reference distance and the centroid of clusters. Specifically, as the state increases, the candidate distance for the near boundary increases, and the candidate distance for the far boundary decreases. The value of the i-th candidate distance for the near and far boundaries (i.e., $nb_{i}$ and $fb_{i}$) is calculated using Equations (11) and (12), respectively.

(11) $$nb_{i}=d_{ref}-c_{m-i}$$

(12) $$fb_{i}=d_{ref}+c_{i-1}$$

where $c_{m-i}$ is the centroid of the (m−i)-th cluster, and $c_{i-1}$ is the centroid of the (i−1)-th cluster. Here, $c_{0}$ is equal to zero. Therefore, $nb_{m}$ and $fb_{1}$ are the same as $d_{ref}$.

To determine ${d^{\prime }}_{nb}$ and ${d^{\prime }}_{fb}$, each Q-learning agent selects the action with the largest Q-value at the current state in the Q-table. If two or more actions have the same largest Q-value, the Q-learning agent randomly selects one of them. The selected actions by each Q-learning agent can be given by Equations (13) and (14), respectively.

(13) $$a_{t}^{nb}=\underset{a}{\arg\;\max}Q(s_{t}^{nb},a)$$

(14) $$a_{t}^{fb}=\underset{a}{\arg\;\max}\hat{Q}(s_{t}^{fb},a)$$

where $s_{t}^{nb}$ and $s_{t}^{fb}$ are the current states for the near and far boundaries, and $a_{t}^{nb}$ and $a_{t}^{fb}$ are the indices of the selected actions for the near and far boundaries, respectively. It is assumed that t is equal to the number of executions of QPZA, and the initial states for both boundaries (i.e., $s_{0}^{nb}$ and $s_{0}^{fb}$) are predefined by the system within $\left\{nb_{1},nb_{2},\cdots ,nb_{m}\right\}$ and $\left\{fb_{1},fb_{2},\cdots ,fb_{m}\right\}$. Upon selecting the action, the next states (i.e., $s_{t+1}^{nb}$ and $s_{t+1}^{fb}$) are determined using Equations (15) and (16).

(15) $$s_{t+1}^{nb}=\left\{\begin{array}{l} s_{t}^{nb}-1\;a_{t}^{nb}=1,\;s_{t}^{nb}>1\\ s_{t}^{nb}+1\;a_{t}^{nb}=3,\;s_{t}^{nb}<m\\ s_{t}^{nb}\;\;\;\mathrm{otherwise} \end{array}\right.$$

(16) $$s_{t+1}^{fb}=\left\{\begin{array}{l} s_{t}^{fb}-1\;a_{t}^{fb}=1,\;s_{t}^{fb}>1\\ s_{t}^{fb}+1\;a_{t}^{fb}=3,\;s_{t}^{fb}<m\\ s_{t}^{fb}\;\;\;\;\mathrm{otherwise} \end{array}\right.$$

Each Q-learning agent then obtains ${d^{\prime }}_{nb}$ and ${d^{\prime }}_{fb}$ as $nb_{s_{t+1}^{nb}}$ and $fb_{s_{t+1}^{fb}}$, respectively. For instance, if the selected action is decrease and $d_{nb}=nb_{3}$ (i.e., $s_{t}^{nb}=3$), ${d^{\prime }}_{nb}=nb_{2}$ (i.e., $s_{t+1}^{nb}=2$).

To adaptively adjust the size of the pending zone, the Q-table is updated based on the reward at every execution of QPZA. The reward indicates the suitability of the selected action for the calculated noise level. Consequently, the value of the reward for each boundary varies based on the difference between the adjusted boundary and the calculated noise level. For example, if the calculated noise level is greater than the size of the adjusted pending zone, the value of the reward for the near boundary (i.e., $r_{t}^{nb}$) will be negative, while that of the far boundary (i.e., $r_{t}^{fb}$) will be positive, thereby expanding the size of the pending zone. The reward calculator calculates the reward by comparing the calculated noise level (i.e., σ) and the adjusted boundaries (i.e., ${d^{\prime }}_{nb}$ and ${d^{\prime }}_{fb}$). Therefore, $r_{t}^{nb}$ and $r_{t}^{fb}$ can be obtained by Equations (17) and (18), respectively.

(17) $$r_{t}^{nb}=\left\{\begin{array}{l} -1\;\;\left|d_{ref}-{d^{\prime }}_{nb}\right|<\sigma /k\\ 0\;\;\;\;\;\left|d_{ref}-{d^{\prime }}_{nb}\right|=\sigma /k\\ +1\;\;\left|d_{ref}-{d^{\prime }}_{nb}\right|>\sigma /k \end{array}\right.$$

(18) $$r_{t}^{fb}=\left\{\begin{array}{l} +1\;\;\left|{d^{\prime }}_{fb}-d_{ref}\right|<\sigma /k\\ 0\;\;\;\;\;\left|{d^{\prime }}_{fb}-d_{ref}\right|=\sigma /k\\ -1\;\;\left|{d^{\prime }}_{fb}-d_{ref}\right|>\sigma /k \end{array}\right.$$

where k is the noise level coefficient.

Once the reward calculator determines the reward, the Q-learning agent updates the Q-table accordingly. Specifically, the Q-table for each boundary is updated using Equations (19) and (20), respectively.

(19) $$Q(s_{t}^{nb},\;a_{t}^{nb})\leftarrow Q(s_{t}^{nb},\;a_{t}^{nb})+\alpha \times \left[r_{t}^{nb}+\gamma \times \underset{a}{\max}Q\left(s_{t+1}^{nb},\;a\right)-Q\left(s_{t}^{nb},a_{t}^{nb}\right)\right]$$

(20) $$Q(s_{t}^{fb},\;a_{t}^{fb})\leftarrow Q(s_{t}^{fb},\;a_{t}^{fb})+\alpha \times \left[r_{t}^{fb}+\gamma \times \underset{a}{\max}Q\left(s_{t+1}^{fb},\;a\right)-Q\left(s_{t}^{fb},a_{t}^{fb}\right)\right]$$

where γ is a discount factor that indicates the significance of future rewards, and α is a learning rate that determines the extent to which the newly obtained Q-value supersedes the old Q-value. Both parameters γ and α are set to values between 0 and 1.

## 4. Implementation and Performance Evaluation

QPZA is designed to enhance the accuracy of RSSI-based proximity classification by adaptively adjusting the size of the pending zone. To evaluate the performance of QPZA, we implemented an RSSI-based proximity classification system with QPZA using Raspberry Pi. We then compared the performance of QPZA with that of an existing scheme that employs a fixed size of the pending zone. The subsequent subsections present the implementation details and the results of the performance evaluation in sequence.

### 4.1. Implementation

The anchor beacon, BLE tag, and BLE tag receiver are implemented on Raspberry Pi 4 Model B, which is equipped with Quad-Core Cortex-A72 1.5 GHz CPU and 2 GB RAM [24] and operates on Raspberry Pi OS. We developed the BLE communication capability of devices operating over the 2.4 GHz ISM band using the BlueZ library [25]. The anchor beacon and BLE tag were set to transmit an advertising packet in the iBeacon packet format every 500 ms, with the TxPower set to −59 dBm. The iBeacon packet format has fixed-length data of 30 bytes containing UUID, major ID, minor ID, and TxPower. The distance estimation model and QPZA model are mounted on the BLE tag receiver. To develop the distance estimation model and QPZA, we collected RSSI data using a database (DB) server built on MySQL. Table 3 shows the specifications of the DB server.

We collected RSSI data in two different environments: a small room (8 × 13 m^2^) and a large room (15 × 21 m^2^). In both environments, the BLE tag receiver was positioned at the center of the room, and the distance between the anchor beacons and BLE tag receiver ($d_{anc}$) was set to 1 m. In the case of the small room, the actual distance between the BLE tags and BLE tag receiver was set to 0.50, 1.00, 1.50, 2.00, 2.50, 3.00, and 4.00 m. In the other case, it was set to 0.50, 1.00, 1.50, 2.00, 2.50, 3.00, 4.00, 5.00, 6.00, and 7.00m. The collected datasets (i.e., LD and NL) were divided into training and test datasets at a ratio of 7:3. Using the LD and NL datasets, we built the linear regression-based distance estimation model and the QPZA using scikit-learn, an open-source Python library [26].

**Figure 4:**
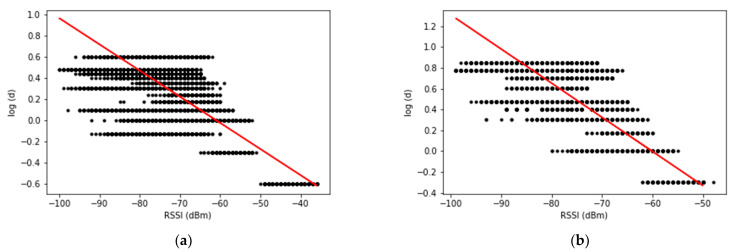
Linear regression models: (**a**) small room; (**b**) large room.

Figure 4a,b depicts the linear regression models that show the relationship between RSSI and log(d) for the small and large rooms, respectively. In the figures, the black dots represent the data in the training dataset of LD, and the red lines indicate the results of Equation (2). In both cases, the value of log(d) decreases as the value of RSSI increases because the signal strength tends to decrease as the distance between the BLE tag receiver and the BLE tag increases. The trained linear regression models are different due to environmental variations such as multi-path fading. Specifically, the parameters A and B in Equation (2) for the small room are −0.02283564 and −1.35625393, and those for the large room are −0.0327501 and −1.96797933, respectively. Consequently, we obtained the linear regression-based distance estimation models for each room, which are expressed by Equations (21) and (22).

(21) $$ed_{sr}=10^{\left(-0.02283564\times RSSI-1.35625393\right)}$$

(22) $$ed_{lr}=10^{\left(-0.0327501\times RSSI-1.96797933\right)}$$

where $ed_{sr}$ and $ed_{lr}$ are the estimated distance for small room and large room, respectively.

To create the deep learning model, we used the Keras and TensorFlow Python open-source libraries [27]. The number of nodes in the input layer was set to five for both rooms, allowing five RSSI inputs to estimate the distance. The number of hidden layers was set to four, with the number of nodes for each hidden layer being set to 64, 64, 32, and 16. The batch size, epoch, and learning rate were set to 200, 200, and 0.0001, respectively.

To create the Q-table used in QPZA, we performed mean-shift clustering on the training dataset of NL. We set the bandwidth for KDE to 0.32 and used a reference distance of 1.5 m and 2.5 m for each room. We obtained three and five clusters for each room, respectively, and the centroids of these clusters were 0.17, 0.33, and 0.50 and 0.22, 0.25, 0.31, 0.38, and 0.57, respectively. Based on these results, we created the states of the Q-tables for each room, as shown in Table 4. We developed the Q-learning agents, including the Q-table, using Java and installed it on the BLE tag receiver.

### 4.2. Performance Evaluation

**Figure 5:**
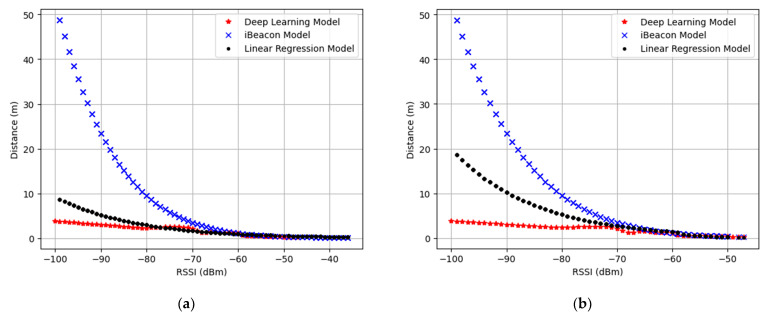
Distance estimation models: (**a**) small room; (**b**) large room.

To evaluate the performance of the distance estimation model, we compared the estimated distance, which varies depending on the models (i.e., the iBeacon, linear regression, and deep learning models). Figure 5a,b shows the distance estimation models for each small and large room, respectively. The iBeacon model is fixed regardless of room type, whereas the linear regression and deep learning models change depending on the training dataset. Therefore, in each figure, an identical iBeacon model is depicted, but different linear regression and deep learning models are displayed. In both figures, the estimated distance tends to exponentially decrease as the RSSI increases, regardless of the estimation model. However, the difference in the estimated distance increases as the RSSI decreases. This is because each model defines the relationship between the RSSI and the estimated distance differently. Specifically, the estimated distance of the iBeacon model is more affected by changes in the RSSI than those of the linear regression and deep learning models are. Comparing the linear regression model and the deep learning model, the latter is less affected by the RSSI.

Table 5 and Table 6 present the estimated distance and root mean square error (RMSE) for the small room and large room, respectively. To obtain the estimated distance, we input the test dataset into both models. The estimated distance for the same actual distance is presented differently in each table since the RSSI dataset used for distance estimation was collected in differently sized rooms. Since the linear regression model is built based on LD, the data value of which varies depending on the environment, it provides more accurate estimation results than the iBeacon model does. Therefore, in the table, the linear regression model has a smaller estimation error (i.e., the difference between the actual distance and the estimated distance) than the iBeacon model does, on average. Consequently, the RMSE of the linear regression model is smaller than that of the iBeacon model. Quantitatively, for the small and large rooms, the linear regression model obtained 76.18% and 65.17% smaller RMSEs compared to the iBeacon model, respectively. Similar to the linear regression model, different deep learning models are built according to the room type. However, the deep learning model uses multiple RSSI inputs to estimate distance; thus, it is less affected by RSSI volatility compared to the linear regression model. Specifically, for each room, the deep learning model has 1.23% and 36.40% smaller RMSEs than the linear regression model does, respectively. The RMSE can be calculated using Equation (23).

(23) $$\mathrm{RMSE}=\sqrt{\frac{\sum_{i=1}^{N}{\left\|y(i)-\hat{y}(i)\right\|}^{2}}{N}}$$

where N is the number of data samples in the test dataset, y(i) represents the actual distance for the i-th data samples, and $\hat{y}(i)$ represents the estimated distance for the i-th data samples.

**Figure 6:**
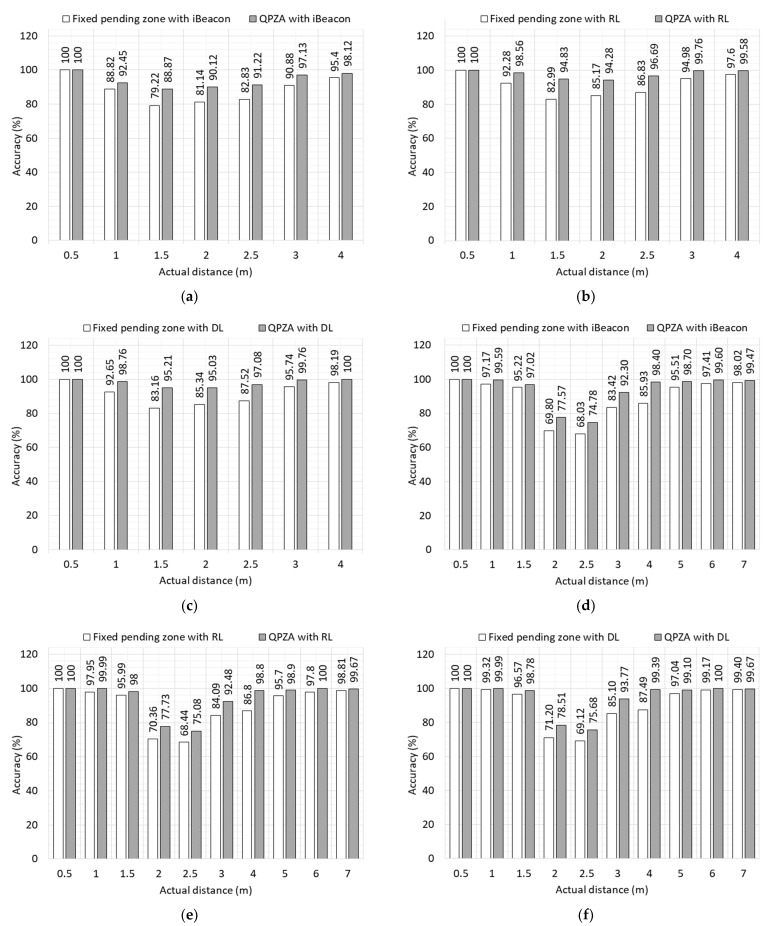
Accuracy of proximity classification for different distance estimation models: (**a**) small room with iBeacon; (**b**) small room with linear regression; (**c**) small room with deep learning; (**d**) large room with iBeacon; (**e**) large room with linear regression; (**f**) large room with deep learning.

To verify the feasibility of QPZA, we compared the accuracy of QPZA with that of the fixed pending zone, the size of which is fixed at 1 m. In the experiment, the accuracy refers to the proportion of correct estimates (i.e., true and false estimates) across all data samples contained in the test datasets. Figure 6a–f shows the accuracy of proximity classification for the small and large rooms using different distance estimation models. The figures indicate that the accuracy reduces when the actual distance is close to the boundaries of the pending zone. This is because the error of the distance estimation model caused by RSSI volatility has a greater effect on proximity classification when the actual distance is closer to the boundaries of the pending zone. In the case of a large room, the error of the average estimated distance was larger compared to that of the small room since the RSSI was more affected by external factors such as multi-path fading when the room size expanded. Therefore, higher accuracy was obtained for the small room compared to the large room. On average, the accuracy of the fixed pending zone and QPZA for a small room was 90.51% and 96.55%, respectively. On the other hand, is the accuracy of these was 89.83% and 94.4% for a large room, respectively. As shown in the figures, the lower the RMSE of the distance estimation model, the higher the average accuracy. Specifically, in the small room, the iBeacon, linear regression, and deep learning models have 88.87%, 90.50%, and 91.14% accuracy, respectively. In the large room, they achieve 93.93%, 95.87%, and 96.69% accuracy, respectively, on average. This is because accurate distance estimation is likely to increase the accuracy of proximity classification. Overall, QPZA showed better performance compared to the fixed pending zone since QPZA adaptively adjusts the size of the pending zone according to the noise level. On average, QPZA has 6.68% and 5.13% higher accuracy than the fixed pending zone does for each room. In the case where the actual distance is the same as the reference distance (i.e., 1.5 m for the small room and 2.5 m for the large room), QPZA achieves 13.67% and 9.70% higher accuracy compared to the fixed pending zone for each room.

**Figure 7:**
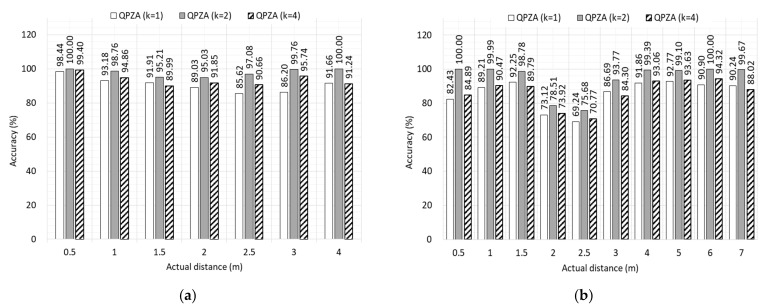
Accuracy of proximity classification for different reward functions: (**a**) small room; (**b**) large room.

To investigate the impact of the reward functions of QPZA on the accuracy of proximity classification, we varied the noise level coefficient (i.e., k in Equations (17) and (18)) between 1, 2, and 4 for each room. Figure 7a,b illustrates the accuracy of proximity classification depending on the noise level coefficient. Regardless of room type, the highest average accuracy of proximity classification was achieved when k=2. This is because the difference between the estimated distance and the reference distance was almost the same as half of the noise level. When k=1, each reward for near and far boundaries was more biased toward –1 and 1, respectively, compared to k=2. Conversely, it was more biased toward 1 and –1, respectively, when k=4. When k=2 and k=4, the pending zone was not appropriately sized, resulting in less accurate proximity classification. Quantitatively, the accuracy of proximity classification for k=2 was 9.10% and 7.34% higher than that for k=1 and k=4, respectively.

## 5. Conclusions

In this paper, we propose QPZA, which aims to improve the accuracy of RSSI-based proximity classification by adaptively adjusting the size of the pending zone based on the noise level of the surrounding environment. To achieve this, QPZA measures the noise level by calculating the difference between the actual distance and the estimated distance of the anchor beacon. The measured noise level is then input into the near and far boundary adjusters, which adjust the near and far boundaries separately. Each boundary adjuster consists of a Q-learning agent and a reward calculator, with the former determining the next boundary using the Q-table and the latter calculating the reward using the measured noise level. The reward is used to update the Q-table of the Q-learning agent. To verify the feasibility of QPZA, we conducted an experimental implementation using Raspberry Pi 4 Model B. We collected the RSSI data of BLE tags and anchor beacons in small and large rooms and developed the distance estimation models and QPZA using the collected datasets and Python’s open-source libraries. We compared the experimental results of QPZA with those of the fixed pending zone, the size of which was set to 1 m. The results showed that QPZA outperformed the fixed pending zone overall, achieving 13.67% and 9.70% higher accuracy of RSSI-based proximity classification compared to the fixed pending zone for the small and large rooms, respectively. In future work, we plan to explore the optimization of proximity classification systems to maximize accuracy outcomes, such as by employing advanced distance estimation models and time series data preprocessing.

## Figures and Tables

**Table 1:** Q-table for near boundary.

Q-Table for Near Boundary Q		Action		
		Decrease	Keep	Increase
State	$nb_{1}$	Q(1,1)	Q(1,2)	Q(1,3)
	$nb_{2}$	Q(2,1)	Q(2,2)	Q(2,3)
	…	…	…	…
	$nb_{m}$	Q(m,1)	Q(m,2)	Q(m,3)

**Table 2:** Q-table for far boundary.

Q-Table for Far Boundary ($\hat{Q}$)		Action		
		Decrease	Keep	Increase
State	$fb_{1}$	$\hat{Q}(1,1)$	$\hat{Q}(1,2)$	$\hat{Q}(1,3)$
	$fb_{2}$	$\hat{Q}(2,1)$	$\hat{Q}(2,2)$	$\hat{Q}(2,3)$
	…	…	…	…
	$fb_{m}$	$\hat{Q}(m,1)$	$\hat{Q}(m,2)$	$\hat{Q}(m,3)$

**Table 3:** DB server specifications.

Component	Description
Operating system	Windows 10 Pro 64 bit
Processor	Intel core i7-8700 CPU 3.20 GHz
HDD	500 GB
RAM	8 GB
Web server	Apache Tomcat
Database	MySQL

**Table 4:** States of near and far boundary for small and large room.

State	Small Room		Large Room	
	Near Boundary (m)	Far Boundary (m)	Near Boundary (m)	Far Boundary (m)
1	1.00	1.50	1.93	2.50
2	1.17	1.67	2.12	2.72
3	1.33	1.83	2.19	2.75
4	1.50	2.00	2.25	2.81
5	-	-	2.28	2.88
6	-	-	2.50	3.07

**Table 5:** Average estimated distance and root mean square error for small room.

Actual Distance (m)	Estimated Distance (m)			RMSE		
	iBeacon	Linear Regression	Deep Learning	iBeacon	Linear Regression	Deep Learning
0.5	0.76	0.86	0.48	0.30	0.37	1.14
1	1.64	1.21	1.03	0.76	0.26	0.92
1.5	2.52	1.36	1.66	1.23	0.18	0.78
2	2.55	1.49	2.36	7.44	1.00	0.70
2.5	7.91	2.59	2.52	2.69	0.53	0.66
3	4.75	2.03	2.59	9.95	1.15	0.66
4	10.22	2.93	2.69	1.43	2.19	0.71
Average	-	-	-	3.40	0.81	0.80

**Table 6:** Average estimated distance and root mean square error for large room.

Actual Distance (m)	Estimated Distance (m)			RMSE		
	iBeacon	Linear Regression	Deep Learning	iBeacon	Linear Regression	Deep Learning
0.5	0.68	0.71	0.44	0.23	0.23	1.18
1	1.59	1.23	1.09	0.74	0.33	0.95
1.5	2.40	1.61	1.54	0.46	0.13	0.79
2	1.91	1.40	2.14	2.84	0.86	0.69
2.5	4.16	2.37	3.51	5.03	1.37	0.63
3	5.68	2.96	2.57	5.27	1.42	0.61
4	6.98	3.48	3.62	1.77	1.04	0.64
5	5.68	3.02	4.29	2.25	2.08	0.71
6	5.82	3.05	6.15	14.39	2.73	0.85
7	18.27	7.66	6.34	4.95	3.01	0.96
Average	-	-	-	3.79	1.32	0.80

## Data Availability

No new data were created or analyzed in this study. Data sharing is not applicable to this article.
